# Rodent heart failure models do not reflect the human circulating microRNA signature in heart failure

**DOI:** 10.1371/journal.pone.0177242

**Published:** 2017-05-05

**Authors:** Eline L. Vegter, Ekaterina S. Ovchinnikova, Herman H. W. Silljé, Laura M. G. Meems, Atze van der Pol, A. Rogier van der Velde, Eugene Berezikov, Adriaan A. Voors, Rudolf A. de Boer, Peter van der Meer

**Affiliations:** 1 Department of Cardiology, University Medical Center Groningen, University of Groningen, Groningen, The Netherlands; 2 European Research Institute for the Biology of Ageing, University of Groningen, Groningen, The Netherlands; National Institutes of Health, UNITED STATES

## Abstract

**Introduction:**

We recently identified a set of plasma microRNAs (miRNAs) that are downregulated in patients with heart failure in comparison with control subjects. To better understand their meaning and function, we sought to validate these circulating miRNAs in 3 different well-established rat and mouse heart failure models, and correlated the miRNAs to parameters of cardiac function.

**Methods:**

The previously identified let-7i-5p, miR-16-5p, miR-18a-5p, miR-26b-5p, miR-27a-3p, miR-30e-5p, miR-199a-3p, miR-223-3p, miR-423-3p, miR-423-5p and miR-652-3p were measured by means of quantitative real time polymerase chain reaction (qRT-PCR) in plasma samples of 8 homozygous TGR(mREN2)27 (Ren2) transgenic rats and 8 (control) Sprague-Dawley rats, 6 mice with angiotensin II-induced heart failure (AngII) and 6 control mice, and 8 mice with ischemic heart failure and 6 controls. Circulating miRNA levels were compared between the heart failure animals and healthy controls.

**Results:**

Ren2 rats, AngII mice and mice with ischemic heart failure showed clear signs of heart failure, exemplified by increased left ventricular and lung weights, elevated end-diastolic left ventricular pressures, increased expression of cardiac stress markers and reduced left ventricular ejection fraction. All miRNAs were detectable in plasma from rats and mice. No significant differences were observed between the circulating miRNAs in heart failure animals when compared to the healthy controls (all P>0.05) and no robust associations with cardiac function could be found.

**Conclusions:**

The previous observation that miRNAs circulate in lower levels in human patients with heart failure could not be validated in well-established rat and mouse heart failure models. These results question the translation of data on human circulating miRNA levels to experimental models, and vice versa the validity of experimental miRNA data for human heart failure.

## Introduction

MicroRNAs (miRNAs) regulate gene expression at the posttranscriptional level by degrading or inhibiting target messenger RNAs (mRNAs). To date, several miRNAs have been described having key roles in cardiac development and cardiovascular disease [1]. Furthermore, several miRNAs have been implicated in the etiology and progression of heart failure [2].

Since the discovery that miRNAs circulate in blood, the search began for new heart failure biomarkers aiding in the diagnosis, prognosis and evaluation of therapy. Previously, we identified a panel of heart failure related circulating miRNAs [3]. These miRNAs were all downregulated in heart failure patients compared to healthy subjects or to patients with respiratory distress due to chronic obstructive pulmonary disease. Further, these miRNAs showed the lowest levels in patients with acute heart failure and a gradual increase towards more stable chronic heart failure and healthy controls, and could be linked to several pathways implicated in the pathophysiology of heart failure [3,4]. These results however contradict the findings of several animal studies of heart failure that report most candidate miRNAs in myocardial tissue to be upregulated, rather than downregulated [5–7]. There are very few data on circulating miRNAs in animal models of heart failure, and both up- and downregulated levels of the differentially expressed miRNAs in the bloodstream have been reported [8,9].

So, while an increasing number of studies identified ever new candidate biomarkers in heart failure patients, few groups conducted experimental follow-up studies in order to gain mechanistic insight into the biology of circulating miRNAs. Animal heart failure models could be useful tools for this purpose and therefore we aimed to investigate an established set of human heart failure related miRNAs in 3 rodent heart failure models.

## Material and methods

### Animals

Animal experiments were performed in accordance with the National Institutes of Health Guide for the Care and Use of Laboratory Animals and were approved by the Animal Ethical Committee of the University of Groningen (permit numbers DEC6954A, DEC6661A and DEC1583-2). Both rats and mice were housed in a 12:12 hour day:night cycle, were monitored regularly and had *ad libitum* access to food and water.

#### Ren2 transgenic rats

Eight homozygous TGR(mREN2)27 (Ren2) male transgenic rats (Max Delbrück Center for Molecular Medicine, Berlin-Buch, Germany) were studied. Homozygous Ren2 rats overexpress the mouse renin-2 gene (ren-2d) and develop severe hypertension and a phenotype of fast forward heart failure within 12–16 weeks, as previously described [10]. Eight age-matched, male Sprague-Dawley (SD) rats were used as control animals (Harlan, The Netherlands).

#### Angiotensin II infused mice

Six 10-week-old male C57Bl/6J mice (Harlan, The Netherlands) underwent subcutaneous administration of angiotensin II (AngII) 2.5 μg/kg per day via osmotic minipumps (Alzet, Palo Alto, CA, USA, model 2004) for 14 days, as previously reported [11]. Six male C57Bl/6J littermates that received subcutaneous saline 0.9% for 14 days served as control group.

#### Ischemic heart failure mice

A total of 8 mice (age 14–16 weeks) underwent permanent ligation of the left coronary artery (LAD) to induce myocardial infarction. Six control mice also underwent surgery but without ligation of the LAD. MRI was performed 4 weeks after the procedure and subsequently hemodynamic measurements were performed. At time of sacrifice, organs were removed and weighed, and blood samples were collected.

### Cardiac magnetic resonance imaging (MRI)

Cardiac MRI measurements have been previously described in more detail [12]. After anesthesia with 2% isoflurane, mice were placed in a vertical 9.4-T, 89-mm bore size magnet equipped with 1500 mT/m gradients and connected to an advanced 400 MR system (Bruker Biospin) using a quadrature-driven birdcage coil with an inner diameter of 3 cm. With the ECG Trigger Unit (RAPID biomedical GmBH), respiration and heart rate were registered. Respiration rate was kept between 20–60 breaths per minute and heart rate between 400–600 beats per minute. Cine MR acquisition and reconstruction was performed with ParaVison 4.0 and IntraGate software (Bruker Biospin GmH). For mice with ischemic heart failure, 8–9 slices were needed for cardiac cine MR images and 7 for control animals. The software QMass (version MR 6.1.5, Medis Medical Imaging Systems) was used to obtain the left ventricular (LV) end-diastolic volume (LVEDV), LV end-systolic volume (LVESV) and LV ejection fraction (LVEF).

### Invasive hemodynamic measurements

Aortic and LV catheterization was performed in all animals as described before [13]. Briefly, after anesthesia with 2% isoflurane an indwelling micromanometer-tipped pressure catheter (0.8 F for AngII and control mice, 1.2 F for ischemic heart failure and control mice and 1.4 F for rats; Millar Instruments, Houston, TX, USA) was inserted into the right carotid artery and advanced into the LV to record intercardiac pressures. Heart rate, aortic pressures, LV end-systolic pressures (LVESP), end-diastolic pressures (LVEDP), first derivative of force (dP/dt_max_) and relaxation constant Tau were measured. dP/dt_max_ values were corrected for peak systolic pressure, as previously reported [10]. Second, after 3 minutes of stabilization, blood pressure measurements were performed in the aortic arch.

### Plasma processing

After the hemodynamic measurements, blood was drawn via cardiac puncture and collected in EDTA tubes. Samples were centrifuged (3000 rpm) for 15 minutes, at 4° Celsius. Plasma was directly stored and frozen in -80° Celsius.

#### MicroRNA measurements in plasma

The miRCURY RNA isolation kit for bodyfluids from Exiqon (Vedbaek, Denmark) was used to isolate RNA from plasma samples of 16 rats and 26 mice. The reversed transcription reactions were performed using the Universal cDNA Synthesis kit (Exiqon). Using a customized Exiqon miRNA PCR panel, the levels of the following circulating miRNAs -previously associated with heart failure-[3] were measured in 50 μl of plasma using a customized Exiqon miRNA PCR panel suitable for rodents; let-7i-5p, miR-16-5p, miR-18a-5p, miR-26b-5p, miR-27a-3p, miR-30e-5p, miR-199a-3p, miR-223-3p, miR-423-3p, miR-423-5p and miR-652-3p. For the miRNA measurements in the ischemic heart failure model we added 2 cardiac-specific miRNAs (miR-208a-3p and miR-499-5p) to our selection. Polymerase chain reactions were conducted with the *LightCycler® 480* (Roche Applied Science, Rotkreuz, Switzerland) with cycle settings as recommended by Exiqon. With use of synthetic RNA templates we controlled for isolation yield, cDNA synthesis and PCR efficiency. Out of a panel of potential reference miRNAs, the miRNAs miR-30a-5p and cel-miR-39-3p for plasma and miR-93-5p for tissue were selected as best performing by GeNorm and NormFinder (GenEx Professional software, MultiD Analyses, Sweden). Detailed information including quality control and normalization of the miRNA measurements is provided in S1 Methods). Expression levels of the measured miRNAs were normalized against the selected reference genes. The delta Ct method was performed to obtain the relative miRNA expression levels using the GenEx Professional software.

### Tissue procurement

After hemodynamic measurements and blood collection, organs were rapidly excised and weighed. The heart was dissected in atria, LV and right ventricle (RV). The LV myocardial tissue was snap frozen in liquid nitrogen and stored at -80° Celsius and used for RNA analyses. Tissue used for miRNA analyses were frozen and powdered before RNA isolation.

#### RNA isolation for microRNA measurements in tissue

Total RNA was isolated using TRIzol (Invitrogen) according to manufacturer’s instructions. RNA quality and quantity was measured by NanoDrop spectrophotometer (ND-1000, Nanodrop Technologies). The same amount of total RNA (500 μg) per sample was used for the cDNA synthesis with miRCURY LNA™ Universal RT cDNA Synthesis Kit (Exiqon).

#### Atrial natriuretic peptide (ANP) and B-type natriuretic peptide (BNP) expression measurements

To assess markers of cardiac wall stress and remodeling, we measured both ANP and BNP in the LV of rats, and ANP in the LV of Ang II mice and controls. Total RNA was extracted from tissue using TRIzol reagent (Invitrogen, Carlsbad, CA, USA) and 0.5 μg total RNA was reverse transcribed to cDNA using the RNeasy Mini kit (Qiagen Inc, Valencia, CA, USA). qRT-PCR was performed using C1000 Thermal Cycler CFX384 Real-Time PCR Detection System (Bio-Rad Laboratories, Veenendaal, The Netherlands). After quantification of mRNA levels (Bio-Rad CFX Manager 2.0), transcript measurements were normalized against the invariant transcript 36B4. Primer sequences used for qRT-PCR analyses are listed in S1 Table.

### Statistical analyses

GenEx Professional software (MultiD Analyses, Sweden) was used for processing the raw miRNA expression data. Other statistical analyses were conducted with R: A Language and Environment for Statistical Computing, version 3.2.0 (R Foundation for Statistical Computing, Vienna, Austria). Results are presented as mean and standard deviation when normally distributed or median and interquartile range when values were non-normally distributed. Student’s t-tests were performed to investigate differences between normally distributed continuous variables and Mann-Whitney U tests for non-normally distributed continuous variables. Correlation analyses were performed between circulating miRNA levels and parameters of cardiac function. Pearson product moment correlation was performed when data was normally distributed, while Spearman correlation analyses were used for non-normally distributed data. Unless otherwise stated, P-values of ≤0.05 were considered significant.

## Results

### Animal characteristics

#### Ren2 and control rats

Mean age at time of sacrificing was 14.4±1.0 weeks for Ren2 rats and 15.0±0.9 weeks for SD controls. Ren2 and control animal characteristics at time of sacrificing including hemodynamic parameters and biomarker expression levels are presented in Table 1. Ren2 rats showed significantly increased LV and lung weights corrected for total body weight. The mean LVEDP and systolic blood pressure were elevated in the Ren2 rats compared to the SD control rats. In addition, LV contractility was reduced as reflected by lower corrected dP/dt_max_ values, and the LV relaxation time constant Tau was significantly increased, all suggestive of progressive heart failure development. Further, expression levels of cardiac stress markers ANP and BNP were both significantly higher in the LV of Ren2 animals.

**Table 1:** Baseline characteristics rats.

Variable	SD	Ren2	P-value
**Animal characteristics** N =	7–8	8	
Body weight (g)	395.0±28.4	365.9±43.0	0.128
LV weight (mg)	955.9±107.4	1389.0±173.7	<0.001
LV/BW ratio (mg/g)	2.4±0.26	3.8±0.36	<0.001
Lung weight (mg)	992.4±106.9	1194.0±310.9	0.120
Lung/BW ratio (mg/g)	2.51±0.31	3.3±0.89	0.048
**Hemodynamic parameters** N =	8	7–8	
Heart rate (beats/min)	319.0±37.4	330.9±25.3	0.471
Systolic blood pressure (mmHg)	108.0±7.0	148.6±17.4	<0.001
LVEDP (mmHg)	9.0±2.3	21.9±4.2	<0.001
dP/dt_max_	62.3±4.1	51.5±4.0	<0.001
Tau (ms)	10.7±1.3	13.2±1.1	0.001
**Cardiac stress markers** N =	8	7	
ANP/36B4	0.04±0.04	1.45±0.51	<0.001
BNP/36B4	0.30±0.11	1.13±0.33	<0.001

Values are presented as mean with standard deviation. SD indicates Sprague-Dawley; LV, left ventricle; BW, body weight; LVEDP, left ventricular end-diastolic pressure; ANP, atrial natriuretic peptide and BNP, B-type natriuretic peptide. dP/dt_max_ represents the maximum rise in LV pressure in early systole corrected for peak systolic pressure.

#### AngII and control mice

The AngII and control mice were sacrificed around 12 weeks of age. Both LV and lung weights were significantly higher in the AngII mice compared to the control mice, reflecting cardiac hypertrophy and lung congestion (Table 2). Further, similar to the Ren2 rats, systolic blood pressure was significantly higher in AngII mice than control mice and although not significant, a trend of an increased LVEDP and LV relaxation parameter Tau was found in the AngII mice compared to their healthy littermates. Moreover, ANP levels were significantly higher in the LV of AngII animals.

**Table 2:** Baseline characteristics AngII mice and controls.

Variable	Control	AngII	P-value
**Animal characteristics** N =	6	6	
Body weight (g)	28.0±2.5	27.3±1.4	0.559
LV weight (mg)	105.0±13.0	136.0±13.1	0.002
LV/BW ratio (mg/g)	3.7±0.3	5.0±0.6	<0.001
Lung weight (mg)	164.8±14.0	199.5±28.6	0.024
Lung/BW ratio (mg/g)	5.9±0.9	7.3±1.2	0.043
**Hemodynamic parameters** N =	4–5	6	
Heart rate (beats/min)	529.4±53.6	527.8±68.3	0.967
Systolic blood pressure (mmHg)	101.5±6.0	123.8±15.2	0.030
LVEDP (mmHg)	9.0±4.3	14.0±8.1	0.322
dP/dt_max_	57.6±9.1	64.9±5.8	0.161
Tau (ms)	7.4±1.5	9.2±2.5	0.257
**Cardiac stress marker** N =	6	6	
ANP/36B4	0.35±0.07	1.70±0.73	0.006

Values are presented as mean with standard deviation. LV indicates left ventricle; BW, body weight; LVEDP, left ventricular end-diastolic pressure and ANP, atrial natriuretic peptide. dP/dt_max_ represents the maximum rise in LV pressure in early systole corrected for peak systolic pressure.

#### Ischemic heart failure mice

Table 3 presents the characteristics of mice 4 weeks after induction of myocardial infarction and mice without permanent ligation of the coronary artery. Induction of myocardial infarction resulted in large infarct sizes (33.8±10.6%) and these mice developed clear signs of heart failure including lower blood pressure, an increased LVEDP and increased LV weights. Furthermore, LV contractility was decreased (as reflected by low dP/dt_max_ values) and LV relaxation impaired. Cardiac MRI showed significantly elevated end-diastolic and end-systolic volumes in ischemic heart failure mice compared to control animals and a severely deteriorated LVEF.

**Table 3:** Baseline characteristics of mice with ischemic heart failure (IHF) and controls.

Variable	Control	IHF	P-value
**Animal characteristics**	6	8	
Body weight (g)	33.1±3.2	34.5±0.9	0.260
LV weight (mg)	127.7±12.1	152.9±18.4	0.013
LV/BW ratio (mg/g)	3.9±0.1	4.4±0.5	0.024
**Hemodynamic parameters**			
Heart rate (beats/min)	475.5±61.4	512.8±65.2	0.300
Systolic blood pressure (mmHg)	106.1±8.4	96.5±6.5	0.002
LVEDP (mmHg)	10.2±2.7	16.4±6.1	0.040
dP/dt_max_	74.6±10.0	63.3±7.6	0.033
Tau (ms)	7.4±0.8	10.8±2.6	0.010
**MRI parameters**			
LVEDV (ml)	73.6±8.9	121±28.1	0.002
LVESV (ml)	37.9±7.9	101.8±33.9	<0.001
LVEF (%)	48.8±6.3	17.4±8.4	<0.001
Infarct size (%)	-	33.8±10.6	-

Values are presented as mean with standard deviation. LV indicates left ventricle; BW, body weight; LVEDP, left ventricular end-diastolic pressure; MRI, magnetic resonance imaging; LVEDV, left ventricular end-diastolic volume; LVESV, left ventricular end-systolic volume and LVEF, left ventricular ejection fraction. dP/dt_max_ represents the maximum rise in LV pressure in early systole corrected for peak systolic pressure.

### Expression levels of circulating miRNAs

**Fig 1:**
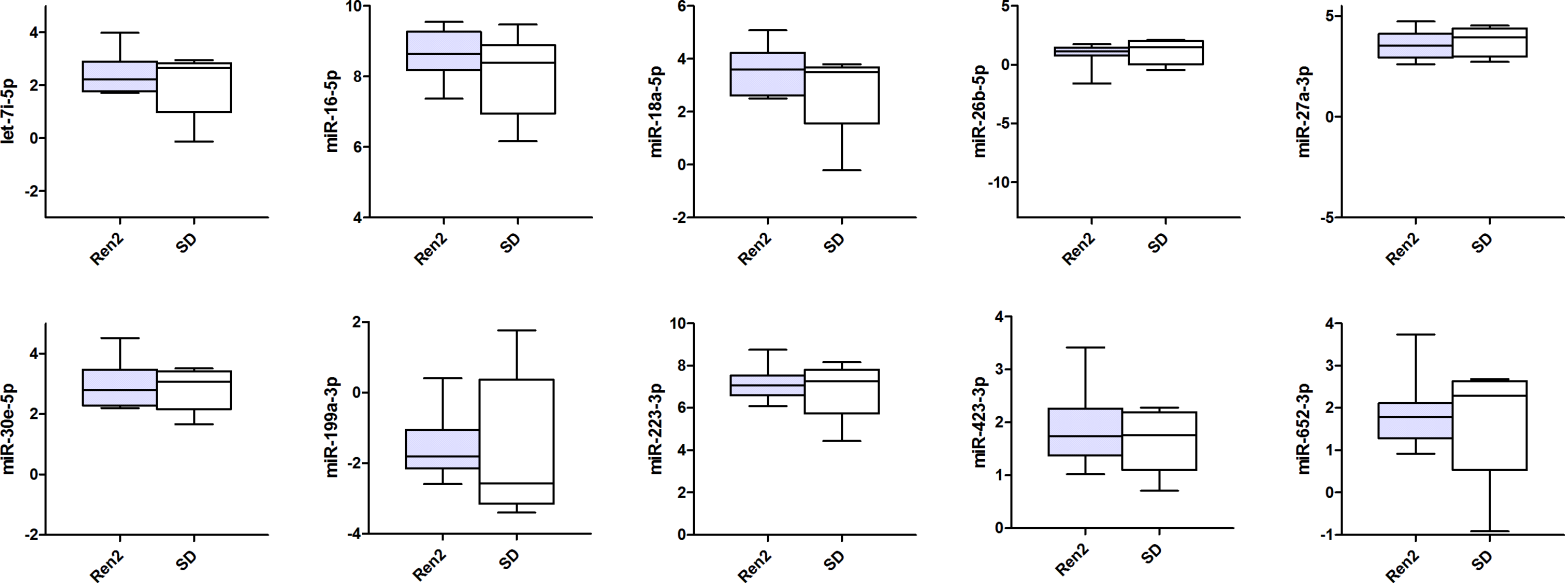
Circulating microRNA expression levels in Ren2 rats and Sprague-Dawley controls (SD). MiRNA expression levels are presented as normalized -Ct values with the median, interquartile range, minimum and maximum values. Differences between groups were not significant for all miRNAs (P>0.05).

**Fig 2:**
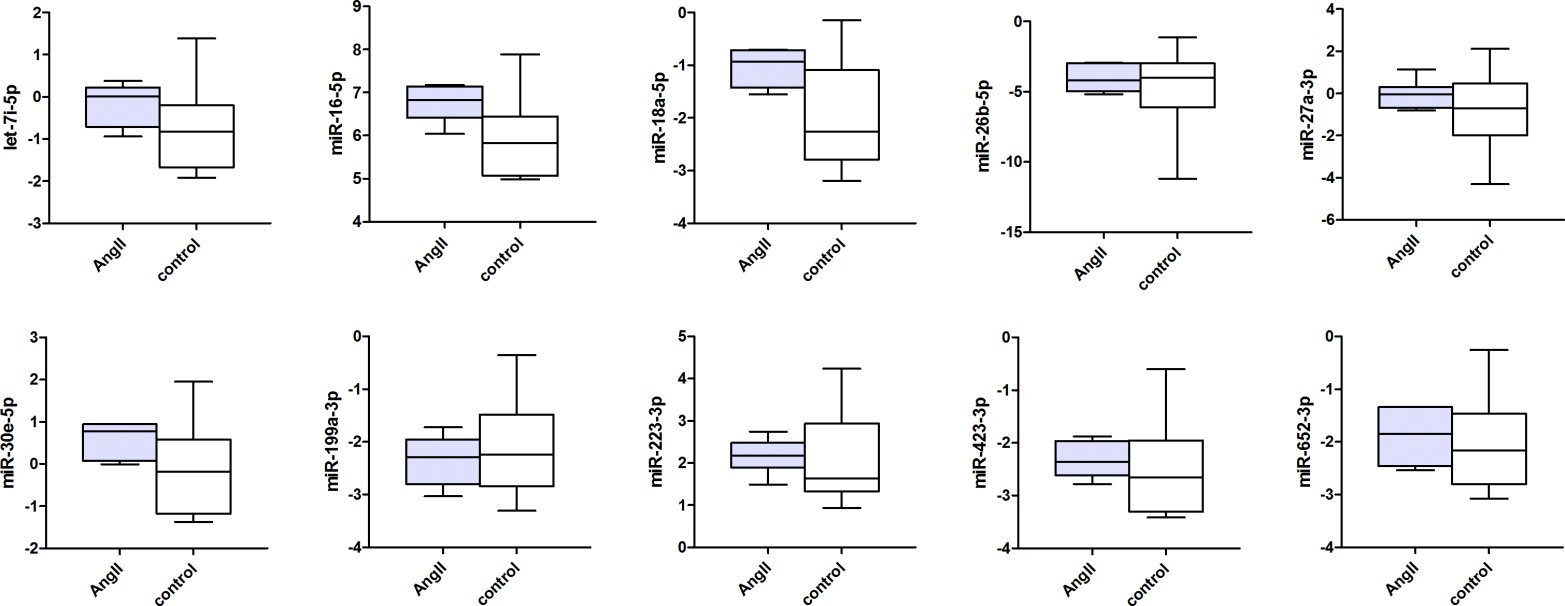
Circulating microRNA expression levels in AngII mice and controls. MiRNA expression levels are presented as normalized -Ct values with the median, interquartile range, minimum and maximum values. Differences between groups were not significant for all miRNAs (P>0.05).

**Fig 3:**
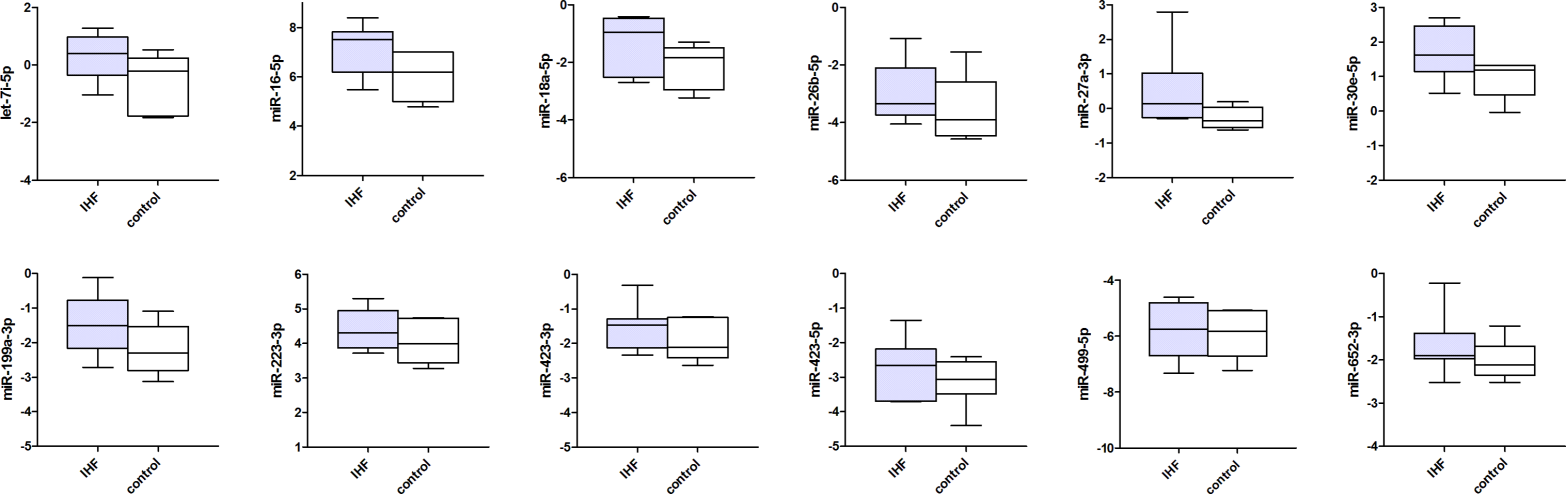
Circulating microRNA expression levels in ischemic heart failure mice and controls. MiRNA expression levels are presented as normalized -Ct values with the median, interquartile range, minimum and maximum values. Differences between groups were not significant for all miRNAs (P>0.05).

Figs 1–3 present the circulating miRNA levels of the detectable miRNAs in the plasma of all 3 different rodent heart failure models. In the Ren2 rats and AngII mice, as well as the ischemic heart failure mice we did not observe any significant differences in plasma miRNA expression levels compared to control animals (all P>0.05, see S2–S4 Tables). Of the cardiac specific miRNAs, miR-208a-3p was not detectable in the plasma of ischemic heart failure mice and miR-499-5p showed the lowest miRNA expression levels in plasma compared to the other miRNAs (Fig 3 and S4 Table).

In general, the rank order of the expression levels of the measured miRNAs was comparable in mice and rats, with the highest miRNA levels of miR-16-5p and miR-223-3p and the lowest levels of miR-199a-3p, miR-652-3p, miR-423-3p and miR-26b-5p (S1–S3 Figs). The majority is in concordance with the rank order of the previously reported miRNA levels in patients with acute- and chronic heart failure and healthy controls [3].

#### Correlations of plasma miRNA levels to parameters of cardiac function

Potential relations between the levels of the circulating miRNAs and parameters of cardiac function (including LVEDP, LVESP, LVEDV, LVESV, LVEF, dP/dt_max_, Tau, ANP and BNP) were investigated using correlation analyses. In rats we found one significant correlation between the normalized -Ct values of miR-423-3p and the dP/dt_max_ values (R = -0.53, P-value = 0.036). In AngII mice and controls, miR-26b-5p was significantly correlated to both LVESP (R = 0.66, P-value = 0.037) and dP/dt_max_ values (R = 0.66, P-value = 0.038) and in mice with ischemic heart failure and controls we found miR-27a-3p to be borderline significantly correlated to LVEF (R = -0.56, P-value = 0.049). However, the above findings did not reach significance after P-value adjustment using Bonferroni correction for multiple testing.

### Expression levels of miRNAs in tissue

To gain more insight in the miRNA expression in tissue, we additionally measured the miRNAs of interest in the LV and kidney of ischemic heart failure mice and controls. In addition to the cardiac specific miR-208a-3p and miR-499-5p, we found that the expression of let-7i-5p, miR-16-5p, miR-27a-3p, miR-199a-3p and miR-223-3p was significantly higher in the heart compared to the kidney, independent of the presence of ischemic heart failure (S4 Fig and S5 Table). No differences in miRNA expression were found in kidneys of ischemic heart failure mice compared to control animals (S6 Table) and only small differences were observed between expression levels of miR-18a-5p, miR-30e-5p, miR-199a-3p and miR-223-3p in the LV of mice with ischemic heart failure compared to controls (S7 Table), however not reaching significance after Bonferroni correction for multiple testing.

## Discussion

In cells, it is well-known that miRNAs exert a gene regulating function by targeting the complementary mRNA, which leads to either degradation or translational repression of the mRNA and hence a disturbed protein production [14]. However, outside cells and in circulating blood the function of miRNAs is largely unknown. The discovery of circulating miRNAs attracted strong attention in several diseases, including heart failure, as it was hypothesized that the circulating miRNAs may be used as a proxy for local production and may potentially be useful as biomarkers or guide for therapy. However, more recent data on circulating miRNAs do not corroborate the changes observed in cardiac tissue. We herein set out to validate the striking observation we recently made that the most differentially expressed circulating miRNAs in human heart failure are downregulated, by measuring this comprehensive set of circulating miRNAs in rats and mice with heart failure. The main conclusions of this study are that 1) the selected miRNAs are detectable in plasma of rats and mice, and that 2) levels of miRNAs do not differ between healthy animals and animals with heart failure, nor correlate with parameters of cardiac function.

One possible reason for the lack of differentially expressed circulating miRNAs in the 3 heart failure models might be the fact that although the selected experimental animal models are well-established and often used in heart failure studies, they might not accurately reflect the human clinical diagnosis of heart failure. Transgenic Ren2 rats develop severe hypertension at an early age and as a consequence cardiac hypertrophy and heart failure [15]. AngII infusion in mice also results in hypertension, hypertrophy and cardiac fibrosis [16]. The animals in both models develop heart failure due to pressure overload and an activated RAS system, while in human heart failure, etiology is more diverse and ischemic etiology is often predominant, as reflected in our ischemic heart failure mouse model. Despite these 3 diverse rodent heart failure models, none of the models showed any differences in circulating miRNA expression levels. However, these models could possibly reflect more accurately the chronic heart failure state than acute heart failure, in which we previously found the most pronounced downregulation of circulating miRNAs. Nevertheless, also in patients with more stable forms of heart failure and chronic heart failure we consistently found lower levels of these circulating miRNAs [3].

As these heart failure animal models only reflect the heart failure syndrome in its purest form, other contributing factors important in human heart failure may not be well-represented (or not extensively enough) to result in a differential miRNA response. It is conceivable that our panel of miRNAs reflect other underlying mechanisms contributing to or coexisting with heart failure. Although we do believe that the previously discovered signature of circulating miRNAs in heart failure patients is strongly associated with the heart failure syndrome [3], it is possible that these miRNAs reflect other, less obvious disease processes or comorbidities. For example, previous work from our group showed a clear link between this panel of miRNAs and atherosclerotic pathophysiological processes such as angiogenesis, endothelial dysfunction and inflammation in heart failure patients [17]. Further, concomitant medication use in heart failure patients such as diuretics, ACE inhibitors and beta-blockers are not taken into account in the current heart failure animal models.

It seems unlikely that the lack of conservation between miRNA expression in humans compared to rodents could have contributed to the discrepancy between the miRNA differences found in human plasma and animals with heart failure. In general, miRNAs are well-conserved between species [18] and also a recent study reported highly similar miRNA expression patterns in different organs of rats and humans [19]. Indeed, the majority of miRNAs we previously identified in human plasma had similar sequences in both mice and rats, except for miR-223-3p (similar between humans and mice, different in rats) and miR-106a-5p (not present in rats and different in mice). However, the regulation of processes controlling the release and uptake of miRNAs into and from the circulation during disease may be different in rodents and humans.

To our knowledge, no other studies measured established circulating miRNAs in human heart failure in heart failure rodent models. In human and rodent pulmonary hypertension, Schlosser et al. compared a circulating miRNA profile previously related to pulmonary hypertension in mice, rats and human plasma [20]. Similar to our study, they found large discrepancies in expression levels between different experimental animal models and human pulmonary hypertension, but also comparable relative rank orders of circulating miRNA levels across these models. The latter suggests that in different species, circulating miRNAs may be regulated in a similar, conserved way.

In S8 Table, we summarize the data on the investigated miRNAs in both *in vitro* or *in vivo* rodent heart failure models and human heart failure studies. This table clearly demonstrates the inconsistencies between tissue expression and circulating miRNA levels as well as differences between research groups. A probable cause for this discordancy and major restriction in circulating miRNA research is the variation in methodology. A plethora of different protocols, arrays and normalization methods is currently available, leading to varying and irreproducible results among different studies. Of note, different sources of circulating miRNAs also contribute to inconsistent findings as it has been shown that expression patterns of miRNAs in whole blood (containing cellular miRNAs) and miRNAs in cell-free plasma do not match in the same individuals [21]. In the current study we used the same material and methods as in our previous study in human heart failure (with the exception of recommended protocol adjustments and the use of primers suitable for rodents), therefore the lack of reproducibility in the 3 animal models caused by the methodology of choice is not probable.

The present findings may have implications for future circulating miRNA studies in heart failure. Results obtained from animal studies cannot be directly translated to the human situation and vice versa. Although the majority of circulating miRNA profiling studies are conducted in human disease, in-depth miRNA studies investigating pathophysiological and molecular mechanisms frequently focus on the tissue of interest using animal models. In our study we were able to detect profound miRNA expression differences in the kidney and heart and although not significant, potentially subtle expression differences in the heart of ischemic heart failure mice and controls. However, the origin and function of circulating miRNAs are still elusive, therefore no direct links can be made from miRNAs in the circulation and the function of the same miRNAs in tissue. In heart failure it has been shown that the most abundant miRNAs in the circulation do not reflect the miRNA signature in the myocardium itself [22]. In line with these results, we found that miR-208a-3p and miR-499-5p -both cardiac specific and highly abundant in the heart [23]- were either undetectable or very lowly expressed in the circulation of mice. This suggests that the most differentially expressed circulating miRNAs do not originate from the myocardium and are most likely derived from blood cells and the endothelium [2]. Although there are examples of miRNA-loaded exosome trafficking from the heart to the circulation [24,25], miRNAs in exosomes only represent a very small proportion of the whole miRNA pool in plasma. Consequently, more research should be directed at increasing our understanding of the biology behind the release and uptake of circulating miRNAs. Various experimental and animal models could be useful for these studies, however circulating miRNA patterns found in human disease should be validated in the animal model of choice to ensure comparability of the miRNA response between species and the reliability of subsequent results.

There are limitations of this work. First, relatively small animal numbers were used in this study. Second, because of the lack of a gold standard regarding the techniques of measuring and normalization of circulating miRNAs, other methodologies may result in slightly different findings. However, the presently reported consistent discordant findings between circulating miRNA profiles in human heart failure and animal models of heart failure may be of great importance for future translational circulating miRNA studies.

To conclude, in this study we were able to detect a set of miRNAs previously related to heart failure in plasma of 3 animal models of heart failure. No circulating miRNA expression differences between heart failure animals and matching controls could be observed as previously identified in human heart failure. This study provides valuable information underlining the complex nature of circulating miRNAs and emphasizes on the challenges in the translation of circulating miRNA profiles between species.

## Supporting information

S1 Method MicroRNA measurements, quality control and normalization. (DOCX)

S1 Table List of the ANP, BNP and 36B4 primers used for qRT-PCR. (DOCX)

S2 Table Circulating microRNA levels in Ren2 transgenic rats and Sprague-Dawley (SD) control rats. MIRNA values represent the median and interquartile range or mean ± standard deviation of the normalized Ct values.
(DOCX)

S3 Table Circulating microRNA levels in AngII mice and controls. MIRNA values represent the median and interquartile range or mean ± standard deviation of the normalized Ct values.
(DOCX)

S4 Table Circulating microRNA levels in ischemic heart failure mice and controls. MIRNA values represent the median and interquartile range or mean ± standard deviation of the normalized Ct values.
(DOCX)

S5 Table Tissue microRNA levels in ischemic heart failure mice and controls. MiRNA values represent the median and interquartile range or mean ± standard deviation of the normalized Ct values in the left ventricle (LV) and kidney of the ischemic heart failure mice and control animals.
(DOCX)

S6 Table Renal microRNA expression in ischemic heart failure mice and controls. MiRNA values represent the median and interquartile range or mean ± standard deviation of the normalized Ct values in the kidney of the ischemic heart failure (IHF) mice and control animals.
(DOCX)

S7 Table Cardiac microRNA expression in ischemic heart failure mice and controls. MiRNA values represent the median and interquartile range or mean ± standard deviation of the normalized Ct values in the left ventricle (LV) of the ischemic heart failure (IHF) mice and control animals.
(DOCX)

S8 Table Differences in microRNA expression between studies in human heart failure, mouse and rat heart failure models. Expression of the investigated miRNAs in heart failure are presented with arrows indicating an upregulation or downregulation compared to the control situation (absence of heart failure).
(DOCX)

S1 Fig Ct values of microRNA expression in plasma of Ren2 and Sprague-Dawley (SD) rats. Boxplots of the Ct values are presented for both Ren2 rats (grey) and SD rats (white) with the median, interquartile range, minimum and maximum value.
(TIF)

S2 Fig Ct values of microRNA expression in plasma of AngII and control mice. Boxplots of the Ct values are presented for both AngII mice (grey) and control mice (white) with the median, interquartile range, minimum and maximum value.
(TIF)

S3 Fig Ct values of microRNA expression in plasma of mice with ischemic heart failure and controls. Boxplots of the Ct values are presented for both mice with ischemic heart failure (grey) and control mice (white) with the median, interquartile range, minimum and maximum value.
(TIF)

S4 Fig Tissue microRNA expression levels in mice with ischemic heart failure and controls. Boxplots of the normalized -Ct values are presented for the left ventricle (LV) of ischemic heart failure (IHF) mice (grey) and controls (white) as well as for kidney tissue of IHF mice (stripes) and controls (white) with the median, interquartile range, minimum and maximum value. * indicates significance (p<0.05) between kidney and LV tissue of both IHF and control animals.
(TIF)
