# Supplementary material for: Rodent heart failure models do not reflect the human circulating microRNA signature in heart failure
Source: PLoS One. 2017 May 5;12(5):e0177242. doi: 10.1371/journal.pone.0177242 (PMC5419653; doi:10.1371/journal.pone.0177242)
Supplement: S1 Method — (DOCX) [file pone.0177242.s001.docx]

**S1 Methods. MicroRNA measurements, quality control and normalization**
A customized miRNA PCR panel from Exiqon was used for the circulating miRNA measurements in the plasma of mice and rats. Several quality control procedures were performed in regards to ensuring the quality of the samples and sample processing. Our samples were processed according to the protocols provided by Exiqon. These approaches were extensively reviewed and evaluated by Blondal et al.[1] Synthetic RNA templates (UniSps) were used to monitor the efficiency of the isolation procedure (UniSp4), cDNA synthesis (UniSp6) and PCR (UniSp3). A negative control cut-off was applied excluding samples which were detected with less than 5 Cps lower than the corresponding negative control assay. Measurements with Ct-values ≥ 37 were excluded from further analyses. All outliers that did not fit the required standards were automatically pinpointed by the GenEx software, provided by Exiqon.

Out of a panel of potential reference miRNAs (based on our previous experiences and as suggested by Exiqon), we used GeNorm and NormFinder (GenEx Professional software, MultiD Analyses, Sweden) to select the best performing reference genes. The miRNAs miR-30a-5p and cel-miR-39-3p were selected for the plasma experiments based on our results from the rat model. Also in mice, these reference miRNAs showed stable expression levels in plasma (Table 1 and Figure 1 and 2) and were selected for the further plasma experiments in mice. For mice tissue, we selected miR-93-5p as best performing normalization miRNA. M-values (average expression stability) of the selected reference miRNAs (Table 2) and variability (Figure 2) are low, indicating stable expression levels in all samples, independent of the condition (heart failure or control).

**Table 1. Descriptive statistics of the Ct values of selected reference miRNAs**

| **Model** |  | **Ct values miR-30a-5p** | | | |  | | **Ct values cel-miR-39-3p** | | | | |  | | **Ct values miR-93-5p** | | | | |  |
| --- | --- | --- | --- | --- | --- | --- | --- | --- | --- | --- | --- | --- | --- | --- | --- | --- | --- | --- | --- | --- |
|  |  | **Mean** | **Median** | **IQR** | **min-max** | |  | | **Mean** | **Median** | **IQR** | **min-max** | |  | | **Mean** | **Median** | **IQR** | **min-max** | |
| Ren2 rats and controls - plasma | | 28.67 | 28.55 | 28.11-29.04 | 26.99-31.14 | |  | | 29.62 | 29.67 | 29.25-29.99 | 28.81-30.42 | |  | |  |  |  |  | |
| AngII mice and controls - plasma | | 28.43 | 28.58 | 27.83-29.06 | 26.06-30.43 | |  | | 25.28 | 25.06 | 24.83-25.49 | 24.66-26.74 | |  | |  |  |  |  | |
| Ischemic mice and controls - plasma | | 29.38 | 29.73 | 29.44-30.24 | 25.84-31.23 | |  | | 27.27 | 27.64 | 27.48-27.73 | 23.64-30.37 | |  | |  |  |  |  | |
| Ischemic mice and controls - tissue | |  |  |  |  | |  | |  |  |  |  | |  | | 20.46 | 20.86 | 19.79-21.51 | 16.03-22.65 | |

The mean, median, interquartile range (IQR), minimum (min) and maximum (max) Ct values are presented for the selected miRNAs used for normalization.

**Table 2. GeNorm M-values of selected reference miRNAs**

| **Model** | **M-value miR-30a-5p** | **M-value cel-miR-39-3p** | **M-value miR-93-5p** |
| --- | --- | --- | --- |
| Ren2 rats and controls - plasma | 0.80 | 0.80 |  |
| AngII mice and controls - plasma | 1.04 | 1.20 |  |
| Ischemic mice and controls - plasma | 0.73 | 0.88 |  |
| Ischemic mice and controls - tissue |  |  | 0.98 |

**Figure 1. Ct values of selected reference miRNAs**


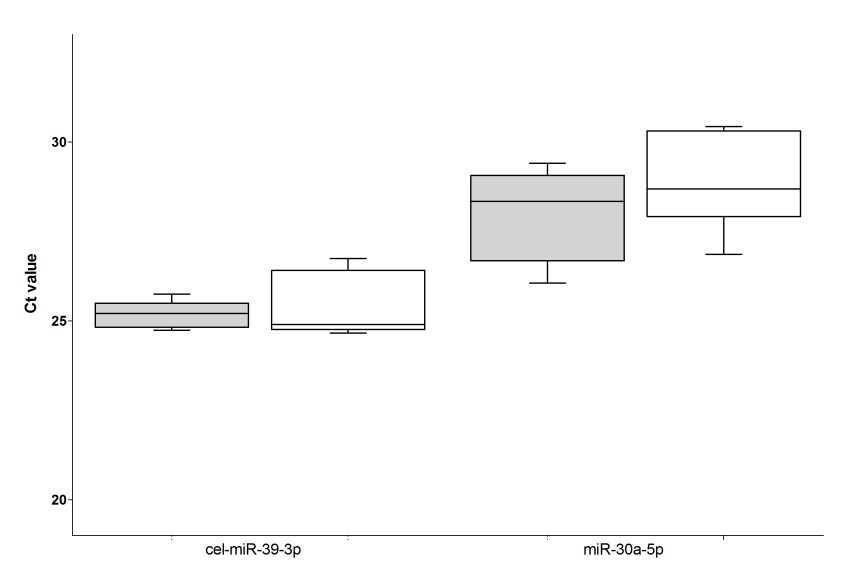

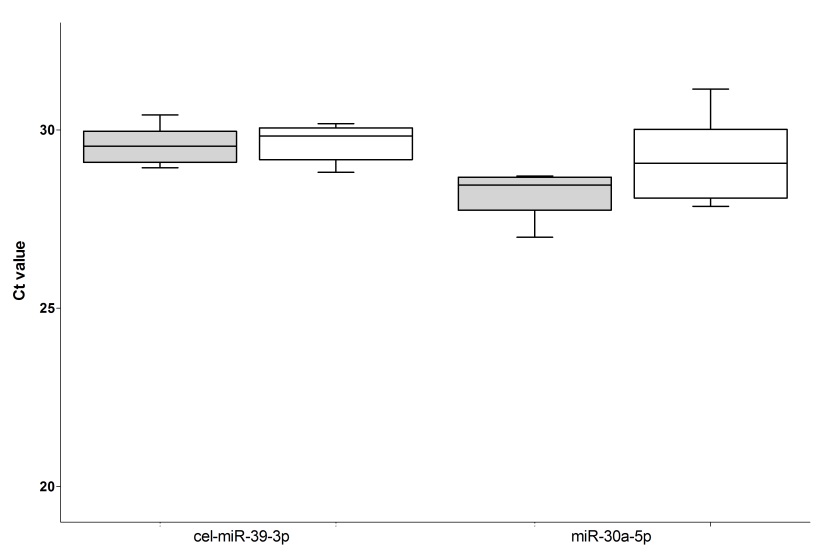


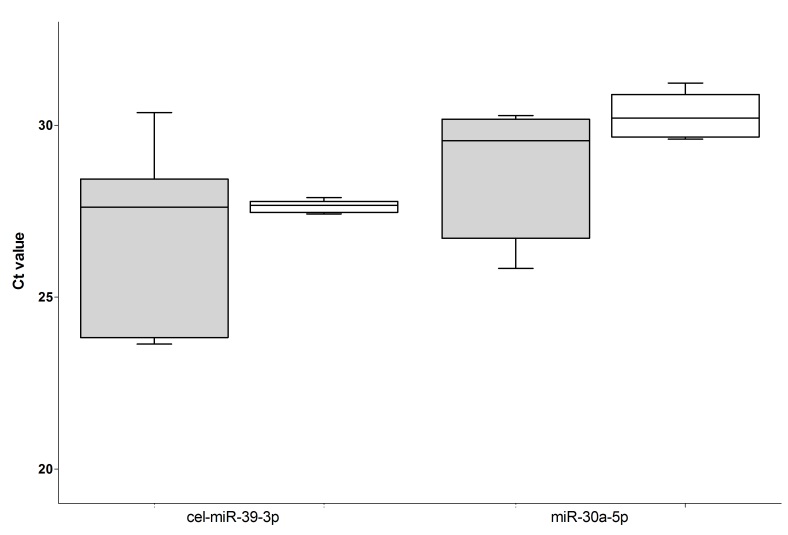


Ct values of miR-30a-5p and cel-miR-39-3p are presented for the Ren2 rats and controls (top left), AngII mice and controls (top right) and ischemic heart failure mice and controls (bottom). Ct values are depicted in boxplots showing the median, interquartile range, minimum and maximum value in heart failure animals (grey) and controls (white).

**Figure 2. NormFinder standard deviation of reference miRNAs**

**
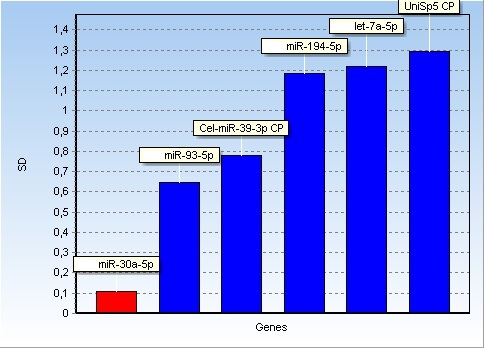

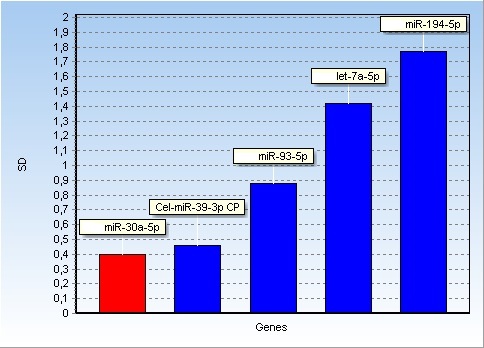
**


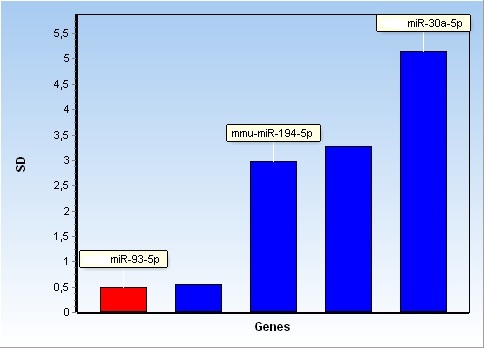


The graphs represent the standard deviation (SD) of potential miRNA reference genes in respectively rat plasma (top graph), mice plasma (middle graph) and mice tissue (bottom graph).

**References**1. Blondal T, Jensby Nielsen S, Baker A, Andreasen D, Mouritzen P, Wrang Teilum M, et al. Assessing sample and miRNA profile quality in serum and plasma or other biofluids. Methods. 2013;59: S1-6.
