# Supplementary material for: Rodent heart failure models do not reflect the human circulating microRNA signature in heart failure
Source: PLoS One. 2017 May 5;12(5):e0177242. doi: 10.1371/journal.pone.0177242 (PMC5419653; doi:10.1371/journal.pone.0177242)
Supplement: S1 Table — (DOCX) [file pone.0177242.s002.docx]

**S1 Table. List of the primers used for qRT-PCR**

| **Name** | **Forward** | **Reverse** |
| --- | --- | --- |
| ANP | ATGGGCTCCTTCTCCATCAC | TCTACCGGCATCTTCTCCTC |
| BNP | ACAATCCACGATGCAGAAGCT | GGGCCTTGGTCCTTTGAGA |
| 36B4 | GTTGCCTCAGTGCCTCACTC | GCAGCCGCAAATGCAGATGG |
