# Supplementary material for: Rodent heart failure models do not reflect the human circulating microRNA signature in heart failure
Source: PLoS One. 2017 May 5;12(5):e0177242. doi: 10.1371/journal.pone.0177242 (PMC5419653; doi:10.1371/journal.pone.0177242)
Supplement: S2 Table — MIRNA values represent the median and interquartile range or mean ± standard deviation of the normalized Ct values. (DOCX) [file pone.0177242.s003.docx]

**S2 Table. Circulating miRNA levels in Ren2 transgenic rats and Sprague-Dawley (SD) control rats**

| **Variable** | **SD** | **Ren2** | **P-value** |
| --- | --- | --- | --- |
| N= | 8 | 8 |  |
| let-7i-5p | -2.6 [-2.8--1.9] | -2.4±0.8 | 1.00 |
| miR-30e-5p | -2.8±0.7 | -2.9±0.8 | 0.78 |
| miR-16-5p | -8.1±1.2 | -8.6±0.7 | 0.27 |
| miR-18a-5p | -3.5 [-3.7--2.8] | -3.5±0.9 | 0.44 |
| miR-223-3p | -6.9±1.3 | -7.2±0.8 | 0.61 |
| miR-652-3p | -2.3 [-2.6--1.5] | -1.9±0.9 | 0.51 |
| miR-423-3p | -1.6±0.6 | -1.9±0.7 | 0.46 |
| miR-26b-5p | -1.2±1 | -1.1 [-1.3--0.9] | 0.44 |
| miR-27a-3p | -3.8±0.7 | -3.6±0.7 | 0.56 |
| miR-199a-3p | 2.6 [1.5-2.9] | 1.5±0.9 | 0.16 |

MiRNA values represent the median and interquartile range or mean ± standard deviation of the normalized Ct values.
