# Supplementary material for: Rodent heart failure models do not reflect the human circulating microRNA signature in heart failure
Source: PLoS One. 2017 May 5;12(5):e0177242. doi: 10.1371/journal.pone.0177242 (PMC5419653; doi:10.1371/journal.pone.0177242)
Supplement: S3 Table — MIRNA values represent the median and interquartile range or mean ± standard deviation of the normalized Ct values. (DOCX) [file pone.0177242.s004.docx]

**S3 Table. Circulating miRNA levels in AngII mice and controls**

| **Variable** | **Control** | **AngII** | **P-value** |
| --- | --- | --- | --- |
| **N =** | **6** | **6** |  |
| let-7i-5p | 0.8 [0.7-1.4] | 0 [-0.2-0.5] | 0.18 |
| miR-30e-5p | 0.2 [-0.1-0.9] | -0.8 [-0.9--0.3] | 0.24 |
| miR-16-5p | -5.8 [-6--5.3] | -6.8 [-7.1--6.6] | 0.07 |
| miR-18a-5p | 2±1.1 | 1±0.4 | 0.09 |
| miR-223-3p | -2.1±1.2 | -2.2±0.4 | 0.85 |
| miR-652-3p | 2.2 [1.9-2.6] | 1.8 [1.4-2.3] | 0.49 |
| miR-423-3p | 2.7 [2.4-3.1] | 2.4 [2.1-2.5] | 0.34 |
| miR-26b-5p | 4 [3.7-4.3] | 4.2 [3.2-4.8] | 0.94 |
| miR-27a-3p | 0.7 [0.2-1.2] | 0.1 [0-0.5] | 0.24 |
| miR-199a-3p | 2.1±1 | 2.3±0.5 | 0.62 |

MiRNA values represent the median and interquartile range or mean ± standard deviation of the normalized Ct values.
