# Supplementary material for: Rodent heart failure models do not reflect the human circulating microRNA signature in heart failure
Source: PLoS One. 2017 May 5;12(5):e0177242. doi: 10.1371/journal.pone.0177242 (PMC5419653; doi:10.1371/journal.pone.0177242)
Supplement: S4 Table — MIRNA values represent the median and interquartile range or mean ± standard deviation of the normalized Ct values. (DOCX) [file pone.0177242.s005.docx]

**S4 Table. Circulating miRNA levels in mice with ischemic heart failure and controls**

| **Variable** | **Control** | **IHF** | **P-value** |
| --- | --- | --- | --- |
| N = | 6 | 8 |  |
| let-7i-5p | 0.7±1.1 | -0.3±0.8 | 0.14 |
| miR-16-5p | -6±1 | -7.1±1 | 0.07 |
| miR-18a-5p | 2.1±0.8 | 1.3±0.9 | 0.14 |
| miR-26b-5p | 3.9 [3.1-4.4] | 3.3 [2.8-3.7] | 0.35 |
| miR-27a-3p | 0.4 [0.1-0.5] | -0.1 [-0.9-0.2] | 0.07 |
| miR-30e-5p | -1±0.6 | -1.7±0.7 | 0.08 |
| miR-199a-3p | 2.2±0.7 | 1.5±0.9 | 0.16 |
| miR-223-3p | -4.1±0.7 | -4.4±0.6 | 0.38 |
| miR-423-3p | 2.1 [1.5-2.3] | 1.5 [1.3-1.8] | 0.49 |
| miR-423-5p | 3.1±0.7 | 2.8±0.9 | 0.45 |
| miR-499-5p | 5.9±0.9 | 5.8±1 | 0.89 |
| miR-652-3p | 2.1 [1.9-2.3] | 1.9 [1.4-2] | 0.30 |

MiRNA values represent the median and interquartile range or mean ± standard deviation of the normalized Ct values.
