# Supplementary material for: Rodent heart failure models do not reflect the human circulating microRNA signature in heart failure
Source: PLoS One. 2017 May 5;12(5):e0177242. doi: 10.1371/journal.pone.0177242 (PMC5419653; doi:10.1371/journal.pone.0177242)
Supplement: S5 Table — MiRNA values represent the median and interquartile range or mean ± standard deviation of the normalized Ct values in the left ventricle (LV) and kidney of the ischemic heart failure mice and control animals. (DOCX) [file pone.0177242.s006.docx]

**S5 Table. Tissue microRNA levels in ischemic heart failure mice and controls**

| **Variable** | **Kidney** | **LV** | **P-value** |
| --- | --- | --- | --- |
| N = | 8 | 8 |  |
| let-7i-5p | -0.8 [-0.9--0.4] | -2.3 [-2.4--2.2] | <0.001 |
| miR-16-5p | -4.7 [-4.7--4.4] | -5.2 [-5.2--5] | 0.001 |
| miR-18a-5p | 4.2 [3.8-4.4] | 4.2 [3.7-4.4] | 0.85 |
| miR-26b-5p | 0.2±0.5 | 0.2±0.6 | 0.88 |
| miR-27a-3p | 0.2 [0.1-0.2] | -1.5 [-1.6--1.4] | 0.002 |
| miR-30e-5p | -2.8±0.5 | -2.9±0.8 | 0.92 |
| miR-199a-3p | 0.1 [0-0.2] | -2.3 [-2.9--1.7] | <0.001 |
| miR-223-3p | 0.7 [0.6-0.9] | -1.7 [-1.8--1.5] | <0.001 |
| miR-423-3p | 1.9 [1.8-2.1] | 2 [2-2.4] | 0.15 |
| miR-423-5p | 3.9 [3.8-4.1] | 4.1 [3.8-4.1] | 0.80 |
| miR-652-3p | 1.4 [1-1.5] | 1.1 [1-1.2] | 0.21 |
| miR-208a-3p | 12.2 [11.5-12.9] | 1.9 [1.3-2.1] | 0.002 |
| miR-499-5p | 11 [10.5-11.7] | -0.5 [-0.6-0.6] | <0.001 |

MiRNA values represent the median and interquartile range or mean ± standard deviation of the normalized Ct values in the left ventricle (LV) and kidney of the ischemic heart failure (IHF) mice and control animals.
