# Supplementary material for: Rodent heart failure models do not reflect the human circulating microRNA signature in heart failure
Source: PLoS One. 2017 May 5;12(5):e0177242. doi: 10.1371/journal.pone.0177242 (PMC5419653; doi:10.1371/journal.pone.0177242)
Supplement: S6 Table — MiRNA values represent the median and interquartile range or mean ± standard deviation of the normalized Ct values in the kidney of the ischemic heart failure (IHF) mice and control animals. (DOCX) [file pone.0177242.s007.docx]

**S6 Table. Renal microRNA expression in ischemic heart failure mice and controls.**

| **Variable** | **Kidney control** | **Kidney IHF** | **P-value** |
| --- | --- | --- | --- |
| N = | 4 | 4 |  |
| let-7i-5p | -0.4 [-0.4--0.3] | -0.9 [-1.2--0.8] | 0.06 |
| miR-16-5p | -4.4 [-4.5--4.4] | -4.8 [-4.8--4.7] | 0.11 |
| miR-18a-5p | 4.4 [4.2-4.4] | 3.8 [3.3-5.6] | 0.70 |
| miR-26b-5p | 0.4±0.3 | 0.1±0.6 | 0.45 |
| miR-27a-3p | 0.2 [0.2-0.5] | 0.1 [-0.3-0.2] | 0.40 |
| miR-30e-5p | -2.6±0.3 | -3.1±0.6 | 0.16 |
| miR-199a-3p | 0.2 [0.1-0.3] | -0.1 [-0.6-0] | 0.20 |
| miR-223-3p | 0.8 [0.7-0.9] | 0.5 [0.1-3] | 0.34 |
| miR-423-3p | 1.9 [1.8-1.9] | 2 [1.6-2.1] | 0.89 |
| miR.423.5p | 3.9 [3.9-4] | 3.8 [3.3-4] | 0.59 |
| miR-652-3p | 1.3 [1-1.5] | 1.4 [1-1.6] | 0.89 |
| miR-208a-3p | 12.2 [11.7-12.7] | 12.4 [11.9-12.8] | 1 |
| miR-499-5p | 11 [10.6-11.3] | 11.3 [10.4-12.2] | 1 |

MiRNA values represent the median and interquartile range or mean ± standard deviation of the normalized Ct values in the kidney of ischemic heart failure (IHF) mice and control animals.
