# Supplementary material for: Rodent heart failure models do not reflect the human circulating microRNA signature in heart failure
Source: PLoS One. 2017 May 5;12(5):e0177242. doi: 10.1371/journal.pone.0177242 (PMC5419653; doi:10.1371/journal.pone.0177242)
Supplement: S7 Table — MiRNA values represent the median and interquartile range or mean ± standard deviation of the normalized Ct values in the left ventricle (LV) of the ischemic heart failure (IHF) mice and control animals. (DOCX) [file pone.0177242.s008.docx]

**S7 Table. Cardiac microRNA expression in ischemic heart failure mice and controls.**

| **Variable** | **LV control** | **LV IHF** | **P-value** |
| --- | --- | --- | --- |
| N = | 4 | 4 |  |
| let-7i-5p | -2.2 [-2.3--2.2] | -2.4 [-2.5--2.4] | 0.23 |
| miR-16-5p | -5.3 [-5.4--5.2] | -5.1 [-5.2--4.9] | 0.18 |
| miR-18a-5p | 4.5 [4.3-4.7] | 3.6 [3.5-3.8] | 0.03 |
| miR-26b-5p | -0.2±0.4 | 0.6±0.6 | 0.07 |
| miR-27a-3p | -1.6 [-1.7--1.5] | -1.5 [-1.6--1.2] | 0.53 |
| miR-30e-5p | -3.5±0.5 | -2.3±0.6 | 0.02 |
| miR-199a-3p | -1.6 [-1.7--1.5] | -3 [-3.4--2.8] | 0.03 |
| miR-223-3p | -1.5 [-1.6--1.5] | -1.8 [-1.9--1.8] | 0.03 |
| miR-423-3p | 2.3 [2-2.9] | 2 [2-2.1] | 0.72 |
| miR-423-5p | 4.1 [3.9-4.1] | 4 [3.9-4.2] | 0.72 |
| miR-652-3p | 1.2 [1.1-1.2] | 1.1 [1-1.1] | 0.20 |
| miR-208a-3p | 1.2 [0.8-1.4] | 2.1 [2.1-2.4] | 0.10 |
| miR-499-5p | -0.7 [-0.9--0.6] | 0.6 [0-1.1] | 0.06 |

MiRNA values represent the median and interquartile range or mean ± standard deviation of the normalized Ct values in the left ventricle (LV) of the ischemic heart failure (IHF) mice and control animals.
