# Supplementary material for: Rodent heart failure models do not reflect the human circulating microRNA signature in heart failure
Source: PLoS One. 2017 May 5;12(5):e0177242. doi: 10.1371/journal.pone.0177242 (PMC5419653; doi:10.1371/journal.pone.0177242)
Supplement: S8 Table — Expression of the investigated miRNAs in heart failure are presented with arrows indicating an upregulation or downregulation compared to the control situation (absence of heart failure). (DOCX) [file pone.0177242.s009.docx]

**S8 Table. Differences in miRNA expression between studies in human heart failure, mouse and rat heart failure models**

|  | **Human heart failure** | | **Rat or mice heart failure models** | |
| --- | --- | --- | --- | --- |
| **miRNA** | Heart tissue | Circulation | Heart tissue/cells | Circulation |
| **miR-18a-5p** | **↓** [1] | **↓** [2], **↑** [3] | **↓** [1] |  |
| **miR-26b-5p** |  | **↓** [2] | **↓** [4,5] |  |
| **miR-423-3p** |  | **↓** [2,6] |  |  |
| **miR-223-3p** |  | **↓** [2] | **↑** [7], **↓** [8,9] | **↑** [10] |
| **miR-199a-3p** | **↑** [11-13], **↓** [14] | **↓** [2,15] | **↑** [11-13,16] |  |
| **miR-27a-3p** |  | **↓** [2,17] | **↓** [4], **↑** [18] |  |
| **miR-652-3p** |  | **↓** [2] | **↑** [19] |  |
| **miR-16-5p** |  | **↓** [2,17] | **↓** [20,21] | **↑** [10] |
| **miR-30e-5p** |  | **↓** [2] | **↓** [22,23] |  |
| **let-7i-5p** |  | **↓** [2] | **↓** [20,24] |  |

Expression of the investigated miRNAs in heart failure are presented with arrows indicating an upregulation or downregulation compared to the control situation (absence of heart failure).

**References**

1. van Almen GC, Verhesen W, van Leeuwen RE, van de Vrie M, Eurlings C, Schellings MW, et al. MicroRNA-18 and microRNA-19 regulate CTGF and TSP-1 expression in age-related heart failure. Aging Cell. 2011;10: 769-779.

2. Ovchinnikova ES, Schmitter D, Vegter EL, Ter Maaten JM, Valente MA, Liu LC, et al. Signature of circulating microRNAs in patients with acute heart failure. Eur J Heart Fail. 2016;18: 414-423.

3. Fang L, Ellims AH, Moore XL, White DA, Taylor AJ, Chin-Dusting J, et al. Circulating microRNAs as biomarkers for diffuse myocardial fibrosis in patients with hypertrophic cardiomyopathy. J Transl Med. 2015;13: 314-015-0672-0.

4. Martinelli NC, Cohen CR, Santos KG, Castro MA, Biolo A, Frick L, et al. An analysis of the global expression of microRNAs in an experimental model of physiological left ventricular hypertrophy. PLoS One. 2014;9: e93271.

5. Han M, Yang Z, Sayed D, He M, Gao S, Lin L, et al. GATA4 expression is primarily regulated via a miR-26b-dependent post-transcriptional mechanism during cardiac hypertrophy. Cardiovasc Res. 2012;93: 645-654.

6. Seronde MF, Vausort M, Gayat E, Goretti E, Ng LL, Squire IB, et al. Circulating microRNAs and Outcome in Patients with Acute Heart Failure. PLoS One. 2015;10: e0142237.

7. Qin D, Wang X, Li Y, Yang L, Wang R, Peng J, et al. MicroRNA-223-5p and -3p Cooperatively Suppress Necroptosis in Ischemic/Reperfused Hearts. J Biol Chem. 2016;291: 20247-20259.

8. Wang YS, Zhou J, Hong K, Cheng XS, Li YG. MicroRNA-223 displays a protective role against cardiomyocyte hypertrophy by targeting cardiac troponin I-interacting kinase. Cell Physiol Biochem. 2015;35: 1546-1556.

9. Shi L, Kojonazarov B, Elgheznawy A, Popp R, Dahal BK, Bohm M, et al. miR-223-IGF-IR signalling in hypoxia- and load-induced right-ventricular failure: a novel therapeutic approach. Cardiovasc Res. 2016;111: 184-193.

10. Dickinson BA, Semus HM, Montgomery RL, Stack C, Latimer PA, Lewton SM, et al. Plasma microRNAs serve as biomarkers of therapeutic efficacy and disease progression in hypertension-induced heart failure. Eur J Heart Fail. 2013;15: 650-659.

11. van Rooij E, Sutherland LB, Liu N, Williams AH, McAnally J, Gerard RD, et al. A signature pattern of stress-responsive microRNAs that can evoke cardiac hypertrophy and heart failure. Proc Natl Acad Sci U S A. 2006;103: 18255-18260.

12. el Azzouzi H, Leptidis S, Dirkx E, Hoeks J, van Bree B, Brand K, et al. The hypoxia-inducible microRNA cluster miR-199a approximately 214 targets myocardial PPARdelta and impairs mitochondrial fatty acid oxidation. Cell Metab. 2013;18: 341-354.

13. Li J, Rohailla S, Gelber N, Rutka J, Sabah N, Gladstone RA, et al. MicroRNA-144 is a circulating effector of remote ischemic preconditioning. Basic Res Cardiol. 2014;109: 423-014-0423-z. Epub 2014 Jul 25.

14. Baumgarten A, Bang C, Tschirner A, Engelmann A, Adams V, von Haehling S, et al. TWIST1 regulates the activity of ubiquitin proteasome system via the miR-199/214 cluster in human end-stage dilated cardiomyopathy. Int J Cardiol. 2013;168: 1447-1452.

15. Ellis KL, Cameron VA, Troughton RW, Frampton CM, Ellmers LJ, Richards AM. Circulating microRNAs as candidate markers to distinguish heart failure in breathless patients. Eur J Heart Fail. 2013;15: 1138-1147.

16. Song XW, Li Q, Lin L, Wang XC, Li DF, Wang GK, et al. MicroRNAs are dynamically regulated in hypertrophic hearts, and miR-199a is essential for the maintenance of cell size in cardiomyocytes. J Cell Physiol. 2010;225: 437-443.

17. Marques FZ, Vizi D, Khammy O, Mariani JA, Kaye DM. The transcardiac gradient of cardio-microRNAs in the failing heart. Eur J Heart Fail. 2016;18: 1000-1008.

18. Nishi H, Ono K, Horie T, Nagao K, Kinoshita M, Kuwabara Y, et al. MicroRNA-27a regulates beta cardiac myosin heavy chain gene expression by targeting thyroid hormone receptor beta1 in neonatal rat ventricular myocytes. Mol Cell Biol. 2011;31: 744-755.

19. Bernardo BC, Nguyen SS, Winbanks CE, Gao XM, Boey EJ, Tham YK, et al. Therapeutic silencing of miR-652 restores heart function and attenuates adverse remodeling in a setting of established pathological hypertrophy. FASEB J. 2014;12: 5097-110.

20. Wang X, Wang HX, Li YL, Zhang CC, Zhou CY, Wang L, et al. MicroRNA Let-7i negatively regulates cardiac inflammation and fibrosis. Hypertension. 2015;66: 776-785.

21. Huang S, Zou X, Zhu JN, Fu YH, Lin QX, Liang YY, et al. Attenuation of microRNA-16 derepresses the cyclins D1, D2 and E1 to provoke cardiomyocyte hypertrophy. J Cell Mol Med. 2015;19: 608-619.

22. Hirt MN, Werner T, Indenbirken D, Alawi M, Demin P, Kunze AC, et al. Deciphering the microRNA signature of pathological cardiac hypertrophy by engineered heart tissue- and sequencing-technology. J Mol Cell Cardiol. 2015;81: 1-9.

23. Roca-Alonso L, Castellano L, Mills A, Dabrowska AF, Sikkel MB, Pellegrino L, et al. Myocardial MiR-30 downregulation triggered by doxorubicin drives alterations in beta-adrenergic signaling and enhances apoptosis. Cell Death Dis. 2015;6: e1754.

24. Satoh M, Minami Y, Takahashi Y, Tabuchi T, Nakamura M. A cellular microRNA, let-7i, is a novel biomarker for clinical outcome in patients with dilated cardiomyopathy. J Card Fail. 2011;17: 923-929.
