# Supplementary figures and images for: Rodent heart failure models do not reflect the human circulating microRNA signature in heart failure
Source: PLoS One. 2017 May 5;12(5):e0177242. doi: 10.1371/journal.pone.0177242 (PMC5419653; doi:10.1371/journal.pone.0177242)

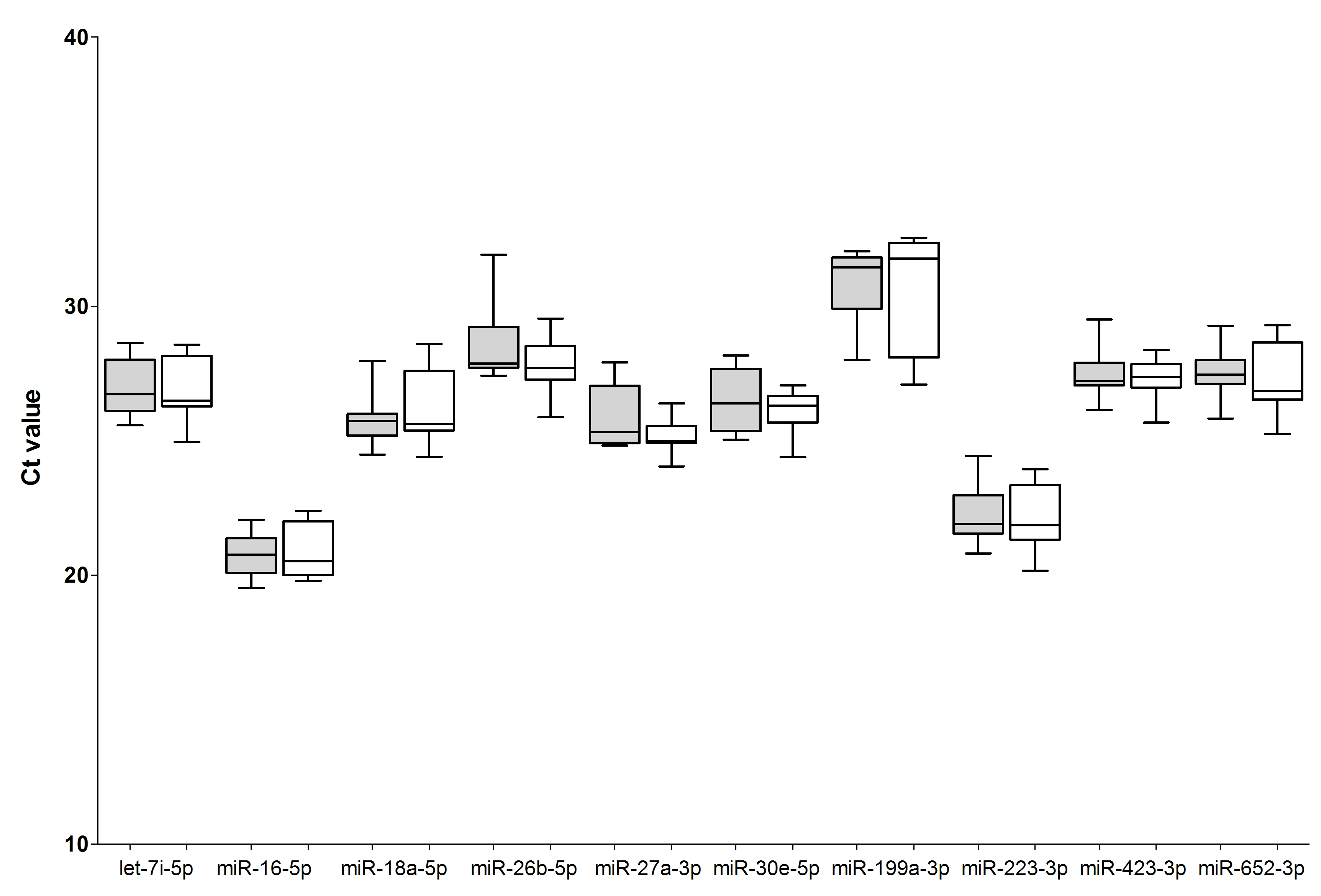

Supplement: S1 Fig — Boxplots of the Ct values are presented for both Ren2 rats (grey) and SD rats (white) with the median, interquartile range, minimum and maximum value. (TIF) [file pone.0177242.s010.tif]

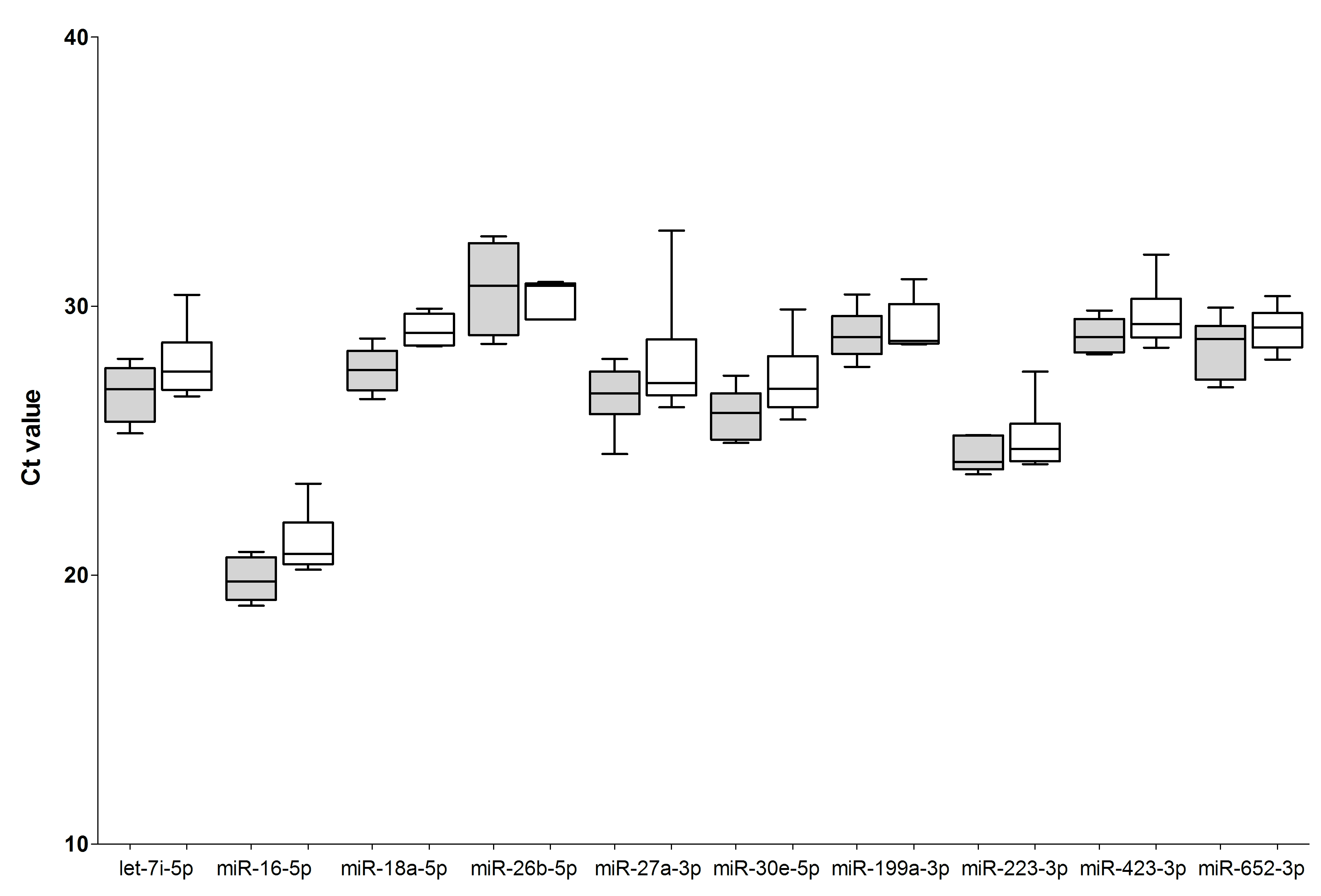

Supplement: S2 Fig — Boxplots of the Ct values are presented for both AngII mice (grey) and control mice (white) with the median, interquartile range, minimum and maximum value. (TIF) [file pone.0177242.s011.tif]

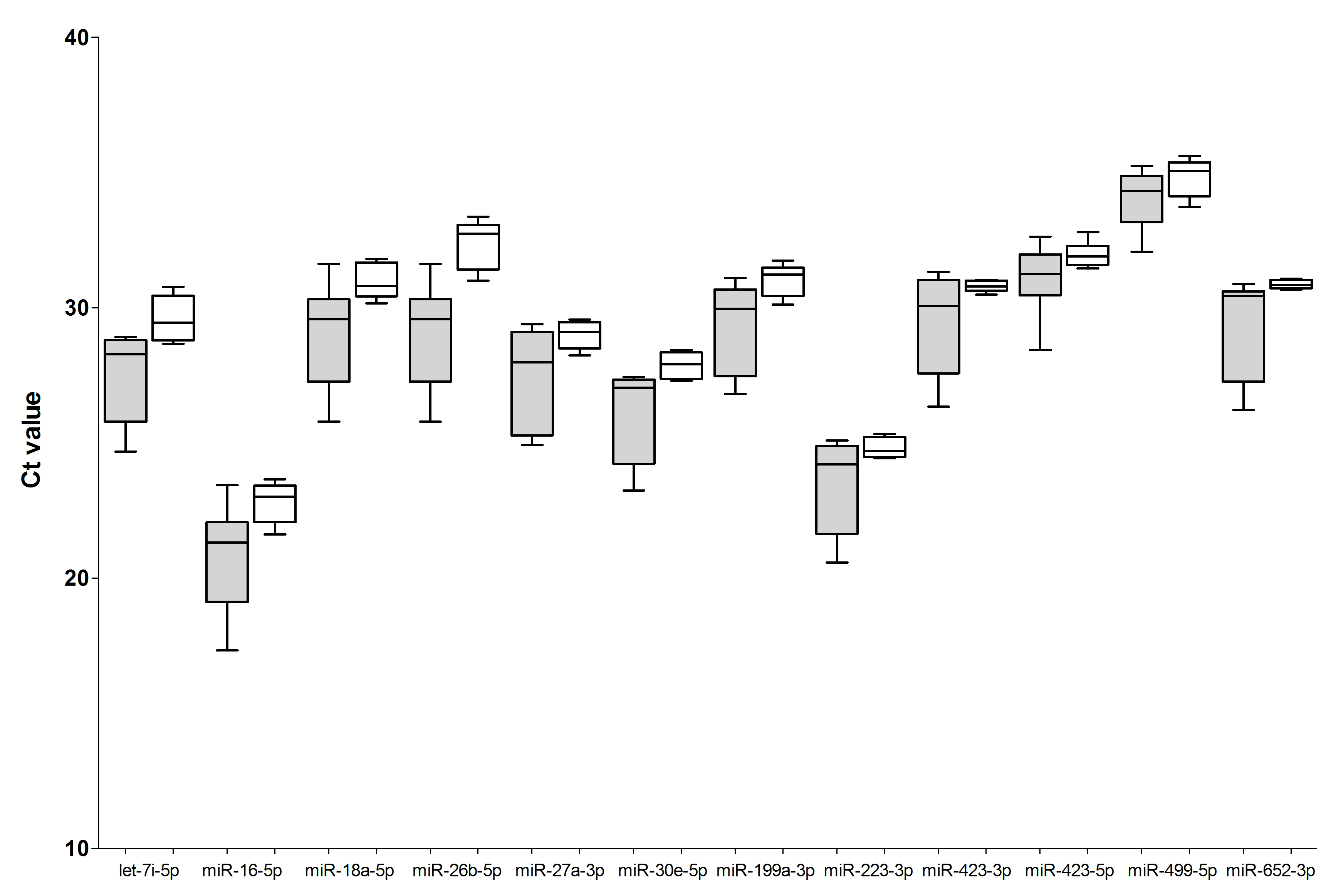

Supplement: S3 Fig — Boxplots of the Ct values are presented for both mice with ischemic heart failure (grey) and control mice (white) with the median, interquartile range, minimum and maximum value. (TIF) [file pone.0177242.s012.tif]

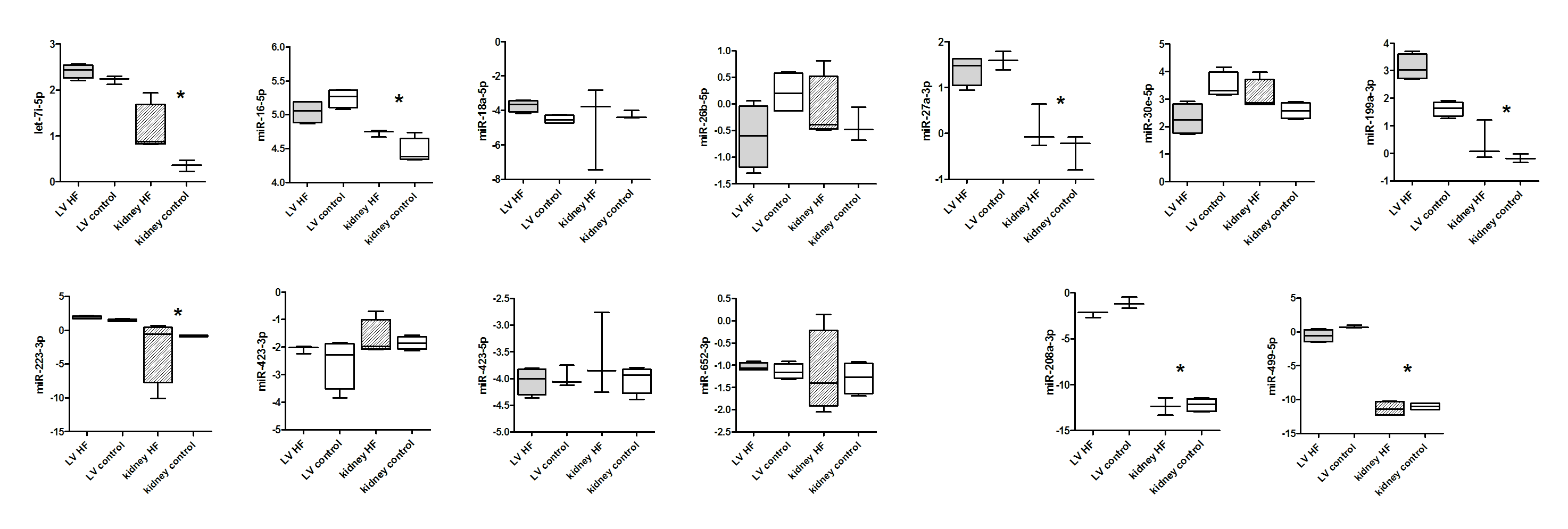

Supplement: S4 Fig — Boxplots of the normalized -Ct values are presented for the left ventricle (LV) of ischemic heart failure (IHF) mice (grey) and controls (white) as well as for kidney tissue of IHF mice (stripes) and controls (white) with the median, interquartile range, minimum and maximum value. * indicates significance (p<0.05) between kidney and LV tissue of both IHF and control animals. (TIF) [file pone.0177242.s013.tif]
